# Designing intracellular metabolism for production of target compounds by introducing a heterologous metabolic reaction based on a *Synechosystis* sp. 6803 genome-scale model

**DOI:** 10.1186/s12934-016-0416-8

**Published:** 2016-01-18

**Authors:** Tomokazu Shirai, Takashi Osanai, Akihiko Kondo

**Affiliations:** RIKEN Center for Sustainable Resource Science, 1-7-22 Suehiro-cho, Tsurumi-ku, Yokohama, Kanagawa 230-0045 Japan; Department of Agricultural Chemistry, School of Agriculture, Meiji University, 1-1-1, Higashimita, Tamaku, Kawasaki, Kanagawa 214-8571 Japan; Organization of Advanced Science and Technology, Kobe University, 1-1 Rokkodaicho, Nada, Kobe, 657-8501 Japan; Department of Chemical Science and Engineering, Graduate School of Engineering, Kobe University, 1-1 Rokkodaicho, Nada, Kobe, 657-8501 Japan

**Keywords:** Genome scale model, Flux balance analysis, Hybrid Metabolic Pathway design (HyMeP), *Synechosystis* sp. 6803, Succinate production

## Abstract

**Background:**

Designing optimal intracellular metabolism is essential for using microorganisms to produce useful compounds. Computerized calculations for flux balance analysis utilizing a genome-scale model have been performed for such designs. Many genome-scale models have been developed for different microorganisms. However, optimal designs of intracellular metabolism aimed at producing a useful compound often utilize metabolic reactions of only the host microbial cells. In the present study, we added reactions other than the metabolic reactions with *Synechosystis* sp. 6803 as a host to its genome-scale model, and constructed a metabolic model of hybrid cells (SyHyMeP) using computerized analysis. Using this model provided a metabolic design that improves the theoretical yield of succinic acid, which is a useful compound.

**Results:**

Constructing the SyHyMeP model enabled new metabolic designs for producing useful compounds. In the present study, we developed a metabolic design that allowed for improved theoretical yield in the production of succinic acid during glycogen metabolism by *Synechosystis* sp. 6803. The theoretical yield of succinic acid production using a genome-scale model of these cells was 1.00 mol/mol-glucose, but use of the SyHyMeP model enabled a metabolic design with which a 33 % increase in theoretical yield is expected due to the introduction of isocitrate lyase, adding activations of endogenous tree reactions via D-glycerate in *Synechosystis* sp. 6803.

**Conclusions:**

The SyHyMeP model developed in this study has provided a new metabolic design that is not restricted only to the metabolic reactions of individual microbial cells. The concept of construction of this model requires only replacement of the genome-scale model of the host microbial cells and can thus be applied to various useful microorganisms for metabolic design to produce compounds.

**Electronic supplementary material:**

The online version of this article (doi:10.1186/s12934-016-0416-8) contains supplementary material, which is available to authorized users.

## Background

Designing optimal intracellular metabolism, as typified by metabolic engineering or synthetic biology, is essential when the aim is mass production of useful compounds by using microbial cells. One of the powerful tools for this design method is the use of genome-scale models (GSMs). In intracellular metabolic reactions, kinetics, of substrates and enzymes are involved, and various mathematical models that take these into consideration have been developed. However, the intracellular metabolism can be assumed to be in a steady state (pseudo-steady state) as long as the cells continue to be in the same environment. In other words, no change in the amount of intermediate metabolites occurs in the cell, and the metabolic flux in the cell can be predicted by mathematical calculation in the form of flux balance analysis (FBA). These computer-calculated prediction results successfully reflect the phenotype of the microbial cells in the actual experiment and have been reported to be highly accurate [1–3]. Many GSMs for different microbial cells have already been developed, and the number is now in excess of 100 (http://gcrg.ucsd.edu/InSilicoOrganisms/OtherOrganisms). Advances in the tools used to organize databases automatically and describe metabolic networks [4] have also led to a rapid increase in the number of new GSMs being developed. Metabolic design tools for various cells have been developed on the basis of these GSMs [5–8]. Using these tools has made it possible to identify the reaction pathways that need to be engineered and achieve high productivity of the target compound with high throughput. Indeed, metabolic prediction tools using GSMs from model microorganisms such as *Escherichia coli*, *Saccharomyces cerevisiae*, and *Corynebacterium glutamicum* have enabled high-efficiency production of compounds such as succinic acid [9], lactic acid [10], lycopene [11], valine [12], vanillin [13], and 1,4-butanediol [14]. In addition, recent years have seen the introduction of omics data such as transcriptomics and metabolomics as parameters for the development of more accurate simulation tools [15–17]. However, metabolic reactions that can be used when performing calculations for the prediction of metabolic flux using GSMs are often confined mainly to reactions that occur in the host cells. For example, when GSMs for *C. glutamicum* or *S. cerevisiae* are used to simulate metabolic design for cells capable of high productivity of useful compounds, it is impossible to automatically use metabolic reactions of the Entner-Doudoroff pathway of *E. coli* or other cells [18]. Moreover, when a simulation is run with *E. coli* as the host, the reaction pyruvic acid → oxaloacetic acid, which is catalyzed by pyruvate carboxylase in *C. glutamicum*, cannot be incorporated into the calculation [19]. Thus, if GSMs only for host microbial cells in actual experiments are considered, it is possible that they end up being limitations to the repertoire of metabolic designs for high productivity of target compounds.

In the present study, we developed a tool with which metabolic reactions that do not belong to a host microorganism can be automatically added to a GSM of that host and simulations can be run. With this tool, individual candidate reactions can be selected from the metabolic reactions in the Kyoto Encyclopedia of Genes and Genomes (KEGG) to construct a computerized metabolic model of a hybrid cell to prepare a new metabolic design with FBA. This tool has been named the Hybrid Metabolic Pathway design tool (HyMeP).

Here, we propose an example of a metabolic design for constructing a HyMeP model for *Synechosystis* sp. 6803 (SyHyMeP) and maximizing the theoretical yield of succinic acid production during glycogen metabolism. *Synechosystis* sp. 6803, which is a model cyanobacterial microorganism, is able to use carbon dioxide as a carbon source during photosynthesis and store glycogen. The complete genome was read in 1996 [20], and many GSMs for it have been developed previously [21–27]. Research on the intracellular metabolism of cyanobacteria and on the production of useful compounds has also advanced in recent years [28–32], and studies have also been conducted on controlling their transcription factors to produce succinic acid during glycogen metabolism [31]. Major advances are expected in the future in research on the production of useful compounds by cyanobacteria. Succinic acid is one of the bio-based 12 building blocks for useful chemical compounds selected by the U.S. Department of Energy (DOE) (http://www.energy.gov/), and, more importantly, it is a compound that is produced from non-fossil raw materials.

## Results and discussion

Design of metabolic pathway for succinic acid production by extended GSM for *Synechosystis* sp. 6803 (SyHyMeP).

In the present study, we used the *Synechosystis* sp. 6803 GSM (SyGSM) developed by Nogales et al. [27]. Metabolic reactions were randomly selected out of all the metabolic reactions obtained from KEGG and added to the SyGSM, and maximum production flux of succinic acid was calculated by FBA. Table 1 shows the numbers of exogenous reactions showing binding to the metabolites in the SyGSM at a particular time. More detailed information is provided in Additional file 1: Table S1. When these were added to the SyGSM and calculations were run to maximize the succinic acid production flux by FBA, we obtained a metabolic design with an improved theoretical yield compared to that with the design when calculations were done only with the SyGSM (Table 2). The focus was on adding reactions where there was a possibility of increase in succinic acid yield. Introduction of isocitrate lyase (KEGG reaction number R00479) was found to be desirable for enabling efficient succinic acid production in *Synechosystis* sp. 6803.

**Table 1:** Number of metabolic reactions linked to the SyGSM, from all metabolic reactions in the KEGG

Number of selected metabolic reactions	Number linked to the SyGSM
1	61
2	26
3	78
4	166
5	245

**Table 2:** Combinations of reactions with improved production yield of succinic acid

Total reaction number (n)	Number of added metabolic pathways (m)	Combinations of metabolic reactions	Rate of increase of succinic acid production yield
0	0		100^*^
1	1	R00479	133
		R00751	102
		R01867	102
2	2	R00751, R00479	144
		R00479, R01867	134
3	3	R00751, R00479, R10179	155

The respective reaction numbers are identical to the KEGG reaction numbers

* The maximum amount of succinic acid produced is 100, as calculated using only the original SyGSM

R00479: Isocitrate → Succinate + Glyoxylate

**Fig. 1:**
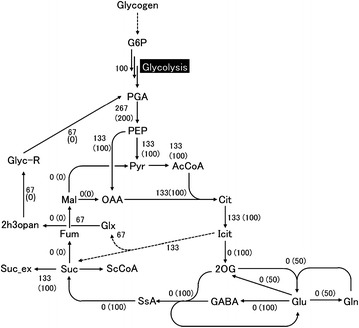
Predicted metabolic flux values when succinic acid production yield increased by 33 %, obtained using SyHyMeP. Flux from glycogen to G6P is 100. Numbers in parentheses are predicted values for intracellular metabolic flux if succinic acid production is maximized using the SyGSM. G6P glucose-6-phosphate; PGA 3-phosphoglycerate; PEP phosphoenolpyruvate; Pyr pyruvate; AcCoA acetyl-CoA; Cit citrate; Icit isocitrate; 2OG 2-oxoglutarate; Glu glutamate; Gln glutamine; GABA γ-aminobutyrate; SsA succinate semialdehyde; Suc succinate; ScCoA succinyl-CoA; Fum fumarate; Mal malate; OAA oxaloacetate; Glx glyoxylate; 2h3opan 2-hydroxy-3-oxopropanoate; Glyc-R D-glycerate

This prediction result is similar to that for metabolic design during succinic acid production in *E. coli* [33–36] or *C. glutamicum* [37, 38]. The existence of fumarate reductases (frdA, B, C, D) as found in *E. coli* and similar organisms has not been confirmed in *Synechosystis* sp. 6803. In fact, the reaction fumarate → succinate has not even been introduced in *Synechosystis* sp. 6803 [21–27]. To that end, succinic acid needs to be produced from citrate in the TCA cycle, and in the original SyGSM, CO_2_ necessarily needs to be discharged. Introducing isocitrate lyase allows carbon to be consumed more sparingly, leading to succinic acid production, and is regarded as a preferred choice in the SyHyMeP as well. *Synechosystis* sp. 6803 does not have a complete TCA cycle, and according to the SyGSM, succinic acid needs to be generated from isocitrate by a GABA-mediated reaction. Introducing isocitrate lyase is therefore regarded as effective because succinic acid can be produced by skipping this relatively long pathway. Another compound that is produced along with isocitrate lyase, that is, glyoxylate, is preferably returned to the glycolytic system by three reactions carried out by *Synechosystis* sp. 6803 (Fig. 1). The succinic acid production yield is potentially increased by up to 33 % due to the introduction of exogenous isocitrate lyase and by the enhanced activity of three reactions that return glyoxylate to the glycolytic system.

R00751 is listed as a candidate reaction for increasing succinic acid production yield.

R00751: l-Threonine → Glycine + Acetaldehyde

The C2 glycine that is generated is returned to the glycolytic pathway via C3 serine by tetrahydrofolate-mediated C1 metabolism, thus allowing for recovery of the carbon. The acetaldehyde that is generated can be returned to the central metabolic pathway, via conversion into acetyl-CoA from acetic acid. Succinic acid yield can also be increased by the reaction R01867.

R01867: (S)-Dihydroorotate + Fumarate → Orotate + Succinate

The SyGSM includes a reaction where orotate and hydrogen peroxide are combined as (S)-dihydroorotate; therefore, functionalizing both reactions would yield a reaction similar to that catalyzed by fumarate reductases of *E. coli* and other organisms. However, this may actually not be practical, because it requires that hydrogen peroxide, which is toxic to the cell, be generated within the cell.

In some GSMs, adding two or more reactions increased succinic acid yield, compared to that in GSMs where only one reaction was added, but these would encompass any of the three reactions mentioned above (Table 2). According to the SyHyMeP, if the theoretical yield of succinic acid production was maximum when R00751 and R00479 are combined, then, it was 155 if a third reaction R10179 was also included. However, it was necessary to activate 19 reactions in *Synechosystis* sp. 6803 besides the three reactions mentioned above, and thus, this design for succinate production is not thought to be realistic (Additional file 2: Table S2). In SyHyMeP, no combination that provided improved yield of succinic acid production was found even when four or five reactions were added.

There have previously been limitations for succinic acid production yield with SyGSM alone. However, introducing the SyHyMeP model has made it possible to automatically add exogenous reactions from other species and to obtain new metabolic designs for improving the production yield of target compounds. It is difficult to produce succinic acid metabolism as designed as shown in Fig. 1, and thus, after introducing the SyHyMeP model, use of algorithms, that take into account both growth and the flux of the target compound by employing tools such as OptForce [39], MOMA [5], and OP-Synthetic [40], is necessary to accomplish a more efficient metabolic design. Moreover, in the actual production of a strain, artificial metabolic switching systems are considered necessary to induce changes in the metabolic flux for growth to produce metabolic flux geared towards the production of the target compound [41–43].

## Conclusions

In the present study, we designed the SyHyMeP, which automatically adds other metabolic reactions from the KEGG to the metabolic reaction model of *Synechosystis* sp. 6803 (SyGSM) and enables new metabolic designs. Thus, new metabolic designs pertaining to succinic acid production during glycogen metabolism in *Synechosystis* sp. 6803 have become possible. The optimal combination of metabolic reactions that could increase the yield of succinic acid production was when isocitrate lyase, represented by KEGG reaction number R00479, was introduced; in this case, three reactions were highly activated via intracellular glyceric acid. The theoretical yield for succinic acid at this time was 133. The HyMeP model proposed here was developed for *Synechosystis* sp. 6803, but the concept of construction of this model requires only replacing the GSM of host microbial cells, and thus can be applied to various microorganisms for developing metabolic designs to produce useful compounds. In other words, it is now possible to enable new metabolic designs that could not be designed with GSMs previously constructed for the metabolic reactions of individual microbial cells.

## Methods

**Fig. 2:**
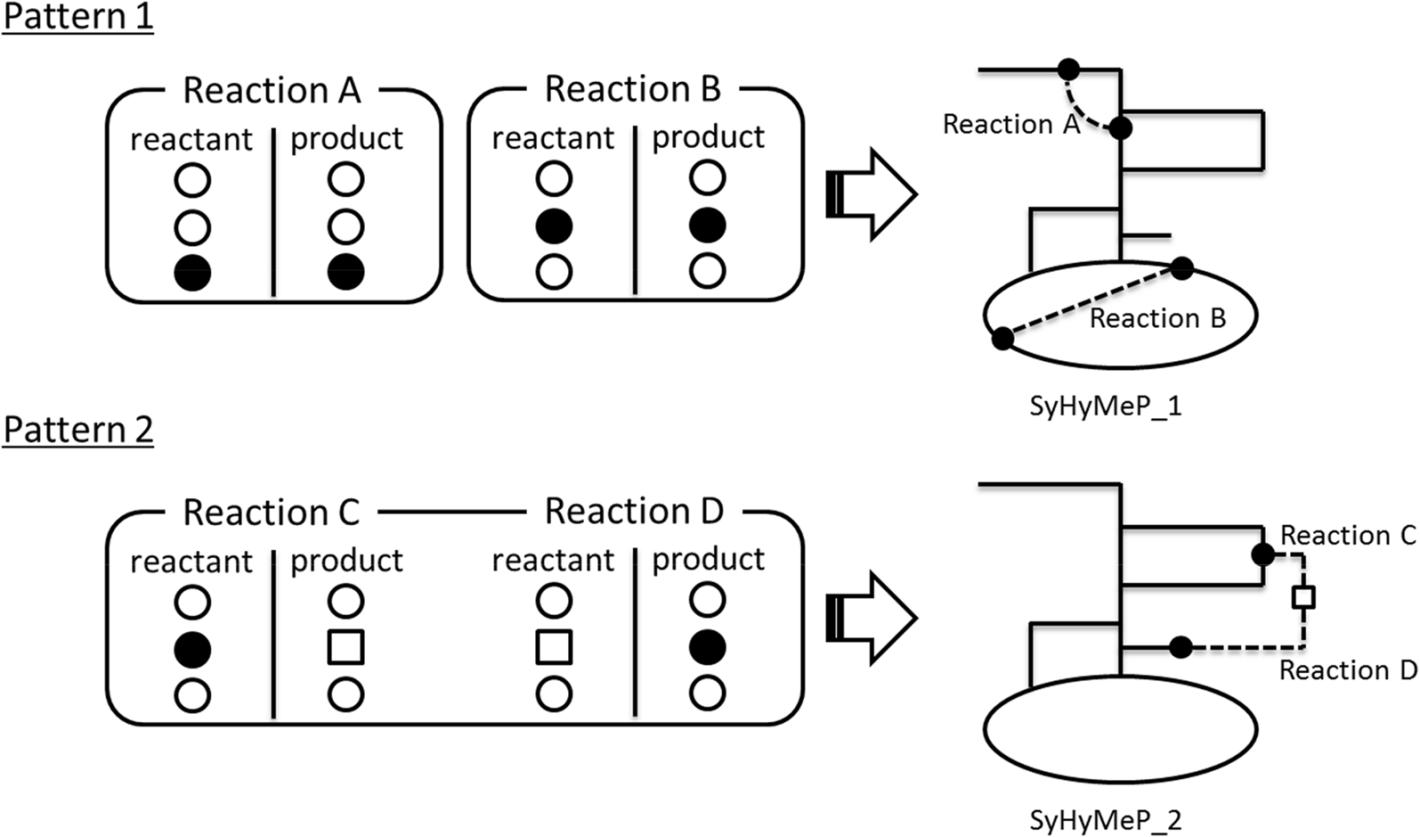
Method for building the SyHyMeP if two metabolic reactions are to be added. In some instances (*Pattern 1*) , the number of metabolic pathways added was two, i.e., metabolic reactions are linked one at a time to the SyGSM, and at other instances (*Pattern 2*), the number of metabolic pathways added was one, i.e., one series of two reactions was linked to the SyGSM. In *Pattern 1*, metabolites present in the SyGSM were included in each of the metabolic reactions (Reactions A and B) (*black symbols*). In *Pattern 2*, Reactions C and D are joined via a metabolite (*square symbol*) not present in the SyGSM, and linked to the SyGSM

The present study is based on the *Synechosystis* sp. 6803 GSM (SyGSM) developed by Nogales et al. [27]. To calculate the succinic acid production flux during glycogen metabolism, the oxygen uptake, and the uptake of CO_2_ from photosynthesis into the cells were set as 0, respectively. SyHyMeP was constructed by using the following procedure: Reactions included in the SyGSM were extracted from the metabolic reaction list provided by the KEGG (http://www.genome.jp/kegg/), and then the other reactions were set as a exSy_list. Inorganic compounds such as H_2_O and ATP and metabolites such as co-factors were excluded (Additional file 3: Table S3, Sy-metabolites) from the metabolites in the SyGSM. These data were used to implement the SyHyMeP as follows: In each metabolic reaction formula in the exSy_list, we determined if metabolites in Sy_metabolites were present in either the Reactants or Products; if any were present, we searched if there were any identical metabolites in the remaining reaction formulae in the exSy_list, with respect to the metabolites on the other side of the reaction. We searched for identical metabolites in either the reactants or products, and if the metabolic reactions were linked, we focused on the metabolite on the other side of the reaction. This procedure was repeated to search and link a specified number of reactions. The SyHyMeP implementation flow diagram is shown in Fig. 2 where, for example, two reactions have been shown to be newly added. At a few instances, the total number of reactions was two; in some cases, single-reaction increments were independently linked to the SyGSM (Fig. 2a), and still in others, each of the reactions was linked (Fig. 2b). Additional file 4: Table S4 (nmlist) summarizes such combinations of up to five reactions. Next, we created a list of reactions that lead to metabolites in the SyGSM in exSy_list (Addditional file 1: Table 1), for each reaction number (maximum reaction number 5). If the total reaction number was set to two, then two reactions (each being n1 in the Additional file 4: Table S4) were combined and added to the SyGSM to prepare the SyHyMeP, and the production yield of succinic acid can be calculated as an FBA (Fig. 2, pattern 1). In addition, each reaction was added to the SyGSM from the list for a reaction number of two (n2 in the Additional file 4: Table S4) to make a separate SyHyMeP, which could be calculated with FBA (Fig. 2, pattern 2).

FBA was used to simulate the metabolic flux distribution in the genome-scale metabolic model [44, 45]. In this study, succinate production was used as the objective function to be maximized.

For metabolic simulation, cytosolic glycogen was used as the sole carbon source, and the uptake rate was set to 100. Other external metabolites such as NH_3_ and CO_2_ could be transported freely through the cell membrane. All simulations were performed using the Java language. SyHyMeP is a web-based application implemented in Java and runs on the Apache Tomcat web server (ver. 7.0.64., http://tomcat.apache.org/). FBA was performed with our original command line tool using OptFlux libraries, which use GLPK (GNU Linear Programming Kit) as a linear programming solver [8].

## Additional files

10.1186/s12934-016-0416-8 List of exogenous metabolic reactions linked to the SyGSM (metabolic reaction number: 1–5).
10.1186/s12934-016-0416-8 Detailed intracellular metabolic reactions that need to be activated, if the yield of succinic acid production according to the SyHyMeP is 155.
10.1186/s12934-016-0416-8 List of metabolites ignored when building the SyHyMeP.
10.1186/s12934-016-0416-8 Total number of metabolic reactions added when building the SyHyMeP, and combinations of metabolic reaction pathways that satisfy them.

## Abbreviations

GSM: genome scale model
FBA: flux balance analysis
HyMeP: Hybrid Metabolic Pathway Design Tool
SyGSM: genome scale model of *Synechosystis* sp. 6803
SyHyMeP: Hybrid Metabolic Pathway design tool of *Synechosystis* sp. 6803
